# Neurological symptoms in adults with Gaucher disease: a systematic review

**DOI:** 10.1007/s00415-024-12439-5

**Published:** 2024-05-21

**Authors:** Gabriele Imbalzano, Claudia Ledda, Alberto Romagnolo, Anna Covolo, Leonardo Lopiano, Carlo Alberto Artusi

**Affiliations:** 1 Department of Neuroscience “Rita Levi Montalcini”, University of Torino, Via Cherasco 15, 10126 Turin, Italy; 2 SC Neurologia 2U, AOU Città della Salute e della Scienza, Corso Bramante 88, 10126 Turin, Italy

**Keywords:** Gaucher disease, Neuronopathic, GBA, Glucosylceramidase, Adult

## Abstract

**Introduction:**

Gaucher disease (GD) is classically divided into three types, based on the presence or absence of neurological signs and symptoms. However, presentation can be highly variable in adulthood, and this aspect has not been adequately addressed in the literature so far. We performed a systematic literature review to analyze the entire spectrum of neurological manifestations in adult patients previously classified as GD type I, II, or III, evaluating the role of variants in different neurological manifestations.

**Methods:**

We searched databases for studies reporting clinical data of adult GD patients (age ≥ 18). Data extraction included GD types, *GBA1* variants, age at disease onset and diagnosis, duration of GD, and age at onset and type of neurological symptoms reported.

**Results:**

Among 4190 GD patients from 85 studies, 555 exhibited neurological symptoms in adulthood. The median age at evaluation was 46.8 years (IQR 26.5), age at neurological symptoms onset was 44 years (IQR 35.1), and age at GD clinical onset was 23 years (IQR 23.4). Parkinsonism, including Parkinson’s disease and Lewy Body dementia, was the most reported neurological manifestation. Other symptoms and signs encompassed oculomotor abnormalities, peripheral neuropathy, seizures, myoclonus, and cerebellar, cognitive and psychiatric symptoms. The genotype N370S/N370S mostly presented with Parkinsonism and the L444P variant with severe and earlier neurological symptoms.

**Conclusion:**

The findings of this systematic review highlight: (1) the relevance of a comprehensive neurological assessment in GD patients, and (2) the importance of considering possible undiagnosed GD in adult patients with mild systemic symptoms presenting unexplained neurological symptoms.

**Supplementary Information:**

The online version contains supplementary material available at 10.1007/s00415-024-12439-5.

## Introduction

Gaucher disease (GD), one of the most common lysosomal storage diseases, results from biallelic variants in the *GBA1* gene located on chromosome 1 (1q21), leading to a defective glucocerebrosidase protein (GCase). This lysosomal enzyme plays a critical role in the metabolism of several glycolipids such as glucosylceramide (GlcCer) and glucosylsphyngosin (GlcSph) [1–4]. The clinical presentation of GD is variable and often involves visceral organs, the bone marrow, and the skeleton, with disease severity ranging from perinatal lethality to asymptomatic cases [1–5]. GD is classified based on the presence (type II and III) or absence (type I) of neurological manifestations. GD type I is the most common form of the disease, accounting for 90–95% of cases in Europe and North America [2, 6–8], with a highly variable clinical onset, from childhood-onset to patients who are asymptomatic throughout their entire life. The neuronopathic types are further categorized as type II or III depending on the acute or chronic nature of the neurological symptoms. Type II GD is characterized by onset in the first years of life, with severe neurological involvement, limited psychomotor development, and a rapidly progressive course to death before the fourth year of life [1, 9–11]. Type III GD patients may also have an early onset, but often have a more slowly progressive course, with longer survival until adulthood [7, 10–12]. Over time, criticism has arisen regarding the distinction among these three phenotypes due to overlapping manifestations and the existence of intermediate phenotypes [12, 13]. Moreover, neurological symptoms, such as parkinsonism, peripheral neuropathy, and nerve root compressions have been described in type I patients [12–16].

The age at diagnosis of GD is variable, depending both on the type of GD and genotype, but it is commonly made in pediatric age [1, 17]. Nonetheless, a diagnostic delay can frequently occur in patients exhibiting only mild or nonspecific systemic symptoms, with unexplained neurological symptoms representing the primary reason for medical evaluation. To date, a comprehensive and systematic evaluation of the multitude of neurological symptoms possibly found in adult GD patients is lacking.

In this context, we conducted a systematic literature review to analyze the broad spectrum of neurological symptoms reported in adult patients with GD. This review aims to provide valuable information for clinicians and researchers concerning the type and frequency of neurological signs and symptoms, genotype–phenotype correlation, and associated clinical and demographic features of patients.

## Materials and methods

### Search method and study selection

**Fig. 1:**
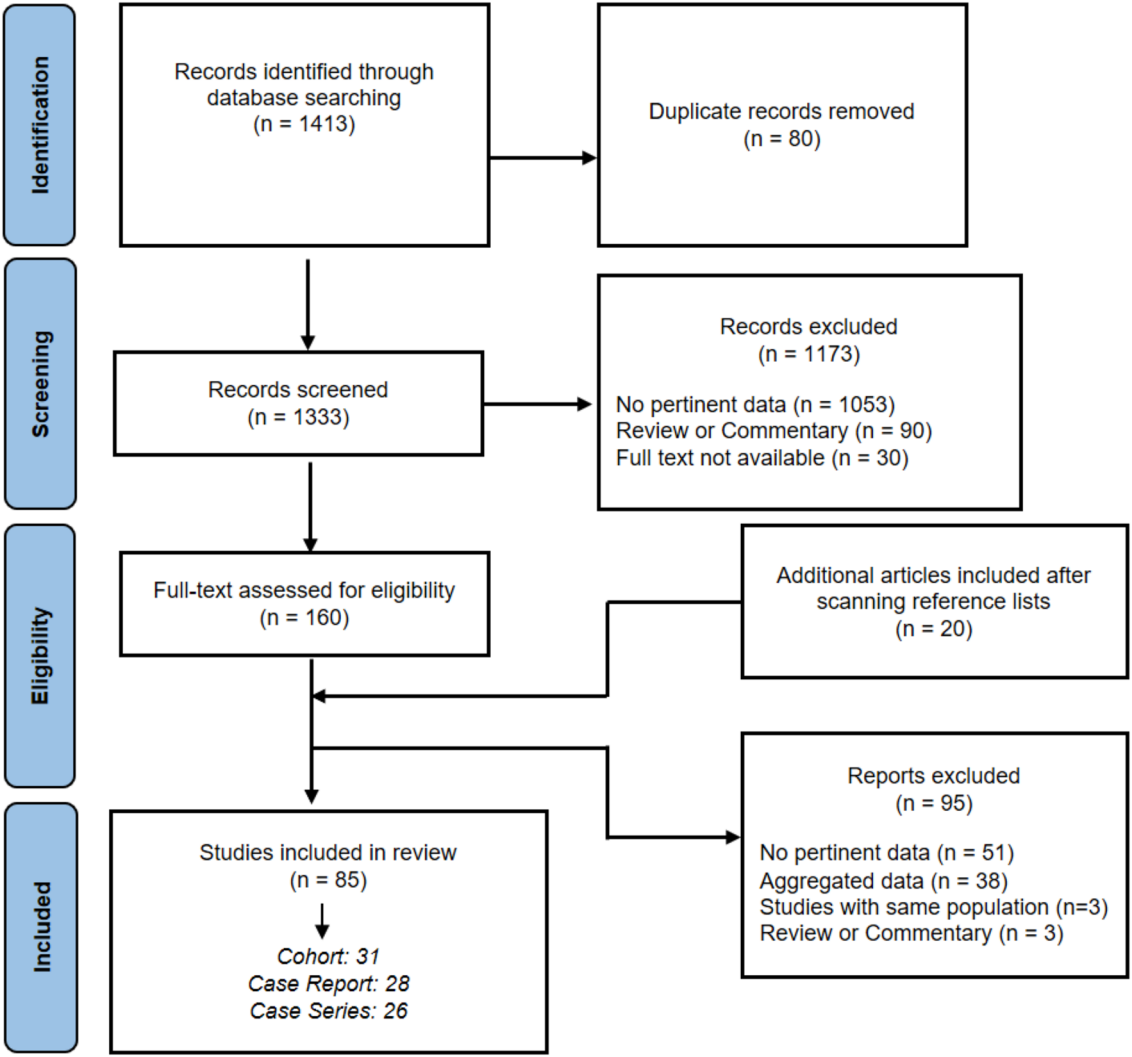
The PRISMA flow diagram of the systematic review

We conducted a systematic review following the Preferred Reporting Items for Systematic Reviews and Meta-analyses (PRISMA) (Fig. 1; Checklist in Supplementary file 1) [18]. We searched PubMed, SCOPUS, and Google Scholar for studies published from January 1st, 1985 to April 1st, 2023, that reported clinical data on GD patients aged 18 and older with one or more neurological symptoms. Only studies referring to human subjects and published in English were considered.

Search strategies are available in Supplementary file 2.

Abstracts and full-text articles were independently reviewed for eligibility criteria by two authors (G.I., C.L.), and the data extraction sheets were checked by two other investigators (C.A.A., A.C.) with independent re-evaluation to solve disagreements, if needed. The reference list of each article was searched to screen for additional pertinent studies not captured by the original search strategy.

We excluded: articles with unavailable full-text, literature reviews, book chapters, editorials, other articles not providing new data, and duplicated studies. Moreover, studies reporting neurological symptoms related to the administration of therapies such as Enzyme Replacement Therapy (ERT) were also excluded (Fig. 1).

### Data extraction

A standardized data collection form was used to extract the following information from each study, when available: number of adults with GD with neurological symptoms; GD type reported; *GBA1* gene variants; age at onset and age at diagnosis of GD; age at onset of neurological symptoms; GD duration and neurological symptoms duration; brain MRI abnormalities.

Included studies were divided as per study design. If two or more studies reported data from the same population, we included the most recent publication with the longest follow-up.

Given the heterogeneity of study designs, the risk of bias of individual studies was evaluated using the National Heart, Lung, and Blood Institute Quality Appraisal Tools as per the Cochrane handbook recommendations [19]. Two reviewers assessed bias independently and a third reviewer was consulted in case of disagreement.

### Data analysis

Results were summarized using mean ± standard deviation (SD) or median (interquartile range – IQR), as appropriate. Included studies were synthesized by major themes, focusing on reported neurological symptoms, and the data were analyzed accordingly. We analyzed the frequency and characteristics of each neurological symptom, providing in the results a specific paragraph for each symptom in order of frequency, and we also reported if more than one neurological symptom was present in a single patient.

### Standard protocol approvals, registrations, and patient consents

The study was registered in PROSPERO (CRD42023412878). Because this was a systematic review of publications, ethical standards committee approval and patient informed consent were not applicable.

## Results

Of 1413 eligible studies found, 85 met the full inclusion criteria (28 case reports, 26 case series, 31 cohort studies) and underwent data extraction and quality assessment (Fig. 1, Table 1). The full reference list can be found in Supplementary file 3.

**Table 1:** Summary of the neurological manifestations evaluated in the literature review

Neurological manifestation	Number of patients affected (total patients included in the studies) – Characterization	Number of studies – Type of studies	Age at evaluation Age at onset of neurological symptoms Age at onset of Gaucher disease Median (IQR)	GBA genotype	Gaucher type
Parkinsonism	297 (416) – 181 Parkinson’s disease (32 cases with poor levodopa response) 11 Lewy Body Dementia 10 complex/atypical parkinsonism 95 undefined parkinsonism	47 studies – 14 case reports 13 case series 20 cohort studies	54.5 (14.9) 51.9 (10.5) 33.7 (17.7)	80 N370S/N370S 36 N370S/L444P 77 N370S/OTHER 8 L444P/OTHER 2 L444P/L444P 3 G377S/G377S 5 OTHER	294 type 1 3 type 3
Oculomotor abnormalities	119 (206) – In 67 patients associated with other neurological symptoms	33 studies – 10 case report 13 case series 10 cohort studies	24 (14) 19.5 (34.1) 13 (15.6)	2 N370S/N370S 2 N370S/L444P 57 L444P/L444P 3 N370S/OTHER 18 L444P/OTHER 5 D409H/D409H 1 G377S/G377S 23 OTHER	39 type 1 1 type 2 78 type 3
Neuropathy	77 (145) – 35 axonal neuropathy 2 mild demyelinating polyneuropathy 19 epidermal denervation at skin biopsy	9 studies – 1 case report 2 case series 6 cohort studies	53 (5.6) NR NR	8 N370S/N370S 10 N370S/L444P 20 N370S/OTHER 2 L444P/OTHER 8 OTHER	77 type 1
Cognitive dysfunction	86 (360) – 63 in the context of parkinsonism	30 studies – 7 case report 9 case series 14 cohort studies	61 (24.5) 51.4 (12.5) 41 (17.9)	20 N370S/N370S 7 N370S/L444P 0 L444P/L444P 15 N370S/OTHER 1 L444P/OTHER 1 G377S/G377S 5 OTHER	80 type 1 6 type 3
Psychiatric symptoms	60 (135) – 51 in the context of parkinsonism	10 studies – 1 case reports 2 case series 7 cohort studies	40 (17.3) 24.4 (33.7) 18 (17)	9 N370S/N370S 4 N370S/L444P 6 L444P/L444P 4 N370S/OTHER 3 L444P/OTHER 8 OTHER	51 type 1 9 type 3
Seizures	48 (192)	23 studies – 8 case reports 7 case series 8 cohort studies	29,7 (10.7) 20 (6) 14 (12.8)	0 N370S/N370S 1 N370S/L444P 11 L444P/L444P 1 N370S/OTHER 3 L444P/OTHER 17 OTHER	10 type 1 38 type 3
Myoclonus	36 (159) – 5 patients in the context of myoclonic epilepsy	17 studies – 6 case reports 5 case series 6 cohort studies	33 (6.4) 22 (17.5) 11 (16.3)	0 N370S/N370S 10 L444P/L444P 1 N370S/OTHER 6 L444P/OTHER 1 G377S/ G377S 9 OTHER	11 type 1 25 type 3
Non-parkinsonian tremor	30 (140)	8 studies – 3 case series 5 cohort studies	NA NA NA	2 L444P/L444P 3 N370S/OTHER 1 OTHER	9 type 1 21 type 3
Cerebellar symptoms	17 (39)	12 studies – 4 case reports 7 case series 1 cohort studies	31.5 (12.6) 17.5 (36.5) 4 (19)	5 L444P/L444P 2 N370S/OTHER 2 L444P/OTHER 2 OTHER	3 type 1 1 type 2 13 type 3
Hypoacusia/hearing loss	22 (134)	8 studies – 4 case series 4 cohort studies	40 (16.5) NR 3,5 (10.6)	2 N370S/L444P 5 L444P/L444P 4 N370S/OTHER 1 OTHER	19 type 1 3 type 3
Mental delay	24 (45)	10 studies – 1 case report 7 case series 2 cohort studies	23,7 (6.7) NR 4,5 (6.4)	14 L444P/L444P 1 L444P/OTHER 8 OTHER	6 type 1 18 type 3
Neurological complications of bone disease	14 (68)	9 studies – 4 case reports 3 case series 2 cohort studies	47 (10.3) 45 (11.5) 22.5 (7.8)	1 N370S/L444P 1 L444P/L444P 1 N370S/OTHER	14 type 1

Abbreviations: *IQR* interquartile range, *NR* not reported, *GBA* Glucocerebrosidase

**Fig. 2:**
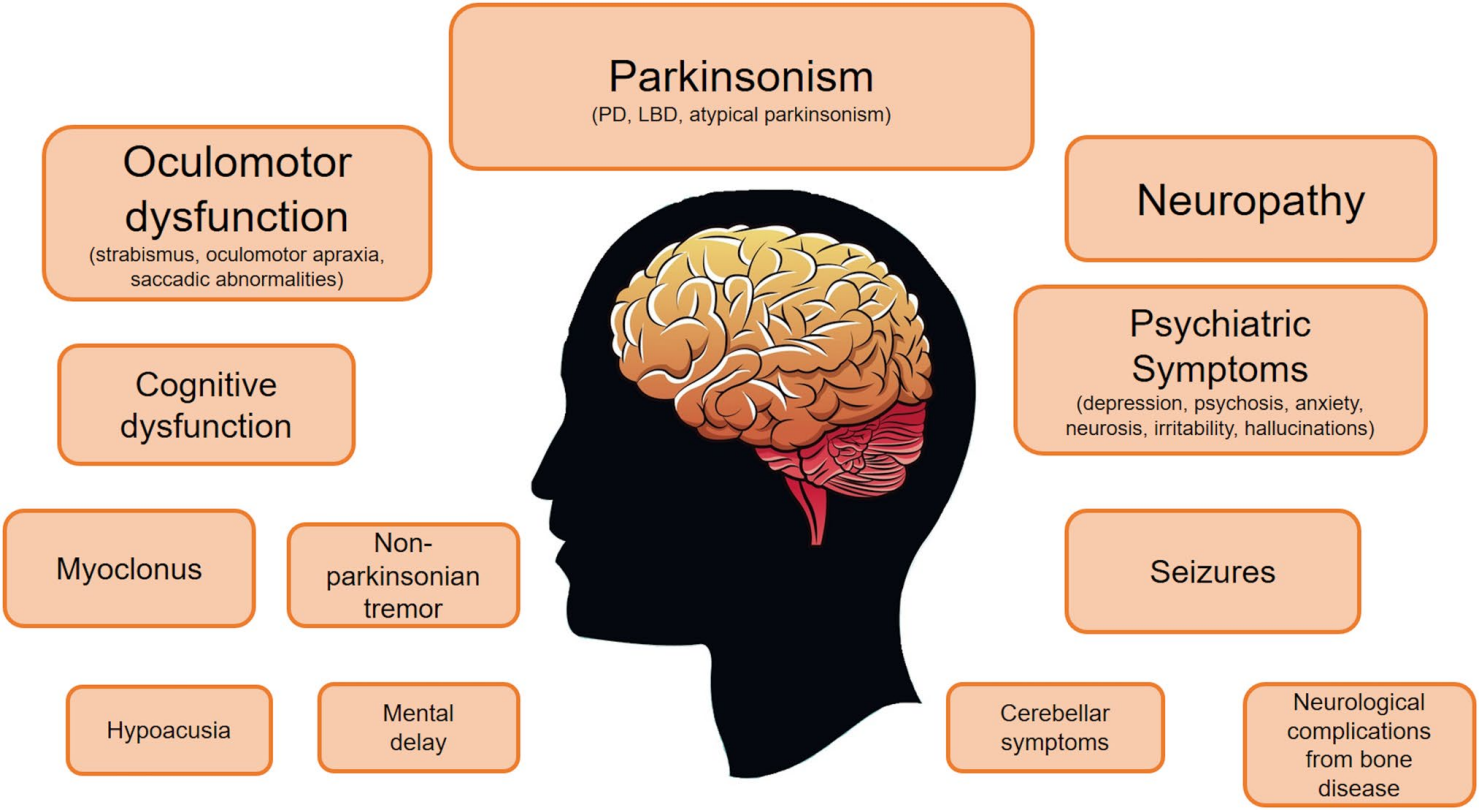
Graphic summary of the principal neurological symptoms evaluated in adult patients with Gaucher Disease. The font size decreases based on the observed prevalence

Included studies provided data from a total of 4190 adult patients with GD, of which 555 presented at least one neurological symptom (sex available for 368 patients, 208 males and 160 females) (Fig. 2). Among them, 447 patients were classified as Gaucher type I, 107 as type III, and one patient as type II. In 411 patients (n = 395 GD type I, and n = 16 GD type III) the neurological symptoms started in adulthood, while in the remaining 144 cases the symptoms were already present before the age of 18, or data about age at onset was not specified.

Considering the entire cohort of 555 patients presenting neurological symptoms, the median age at evaluation, obtained from 78 studies reporting this type of data, was 46.8 years (IQR 26.5), age at onset of neurological symptoms (from 71 studies) was 44 years (IQR 35.1), and age at clinical onset of GD (from 58 studies) was 23 years (IQR 23.4).

Considering the cohort of 411 patients with neurological symptoms described only in adulthood, the median age at evaluation, obtained from 65 studies reporting this type of data, was 50.1 years (IQR 22.4), age at onset of neurological symptoms (from 60 studies) was 47.9 years (IQR 23.2), and age at clinical onset of GD (from 50 studies) was 28.4 years (IQR 23.5).

MRI data were available only for 18 patients from 14 studies, comprising 8 case reports and 6 case series, but with poor characterization, hindering the ability to conduct further analyses.

### Parkinsonism

Parkinsonism was diagnosed in 297 patients from 47 studies evaluating 416 adult GD patients with neurological symptoms (Table 1). This category encompassed 181 cases of Parkinson’s disease (PD), 11 cases of Lewy Body dementia (LBD), 105 of undefined parkinsonism (in 10 cases a complex or atypical parkinsonism was reported, but no additional characterization was provided).

24.2% of patients with Parkinsonism (n = 72/297) were reported to have associated psychiatric or cognitive features (n = 61/297, excluding the 11 cases of LBD); 34.3% of patients (n = 102/297) showed other additional neurological symptoms, encompassing oculomotor abnormalities (11 cases), myoclonus (7 cases), seizures (5 cases), and neuropathy (6 cases). The information regarding the clinical phenotype of PD was scarce, although 32 cases were reported to have a poor levodopa response. Parkinsonism was treated with surgical therapy in 17 GD patients, specifically with pallidotomy in 5 cases, thalamotomy in 3 cases, Deep Brain Stimulation (DBS) of unspecified target in 8 cases, and one case report of bilateral DBS of subthalamic (STN) nuclei. Inefficacy was reported for all cases of pallidotomy and thalamotomy except for one patient, which obtained transient efficacy from pallidotomy. Efficacy from DBS on motor fluctuations of PD was reported in two patients from a case series [20], prolonged for three years in the case report of STN-DBS.

294 patients were diagnosed with GD type I, 3 patients with GD type III. From 211 patients with available genotype, 80 were N370S/N370S, 36 N370S/L444P, 2 L444P/L444P, 77 N370S/Other variant, 8 L444P/Other variant, 3 G377S/G377S, 5 with other variants.

### Oculomotor abnormalities

Oculomotor abnormalities were diagnosed in 124 patients from 33 studies evaluating 206 adults GD patients with neurological symptoms (Table 1). From the studies in which details were provided, we found 8 cases of strabismus, 20 cases of supranuclear gaze palsy, 12 cases of oculomotor apraxia, and 75 cases of saccadic abnormalities (mostly horizontal saccades). Few cases were reported with follow-up over time, and a worsening of the oculomotor abnormalities was described.

54.1% of patients (n = 67/124) presented oculomotor abnormalities associated with other neurological symptoms, encompassing myoclonus (22 cases), neuropathy (2 cases), cerebellar symptoms (10 cases), mental delay (17 cases), Parkinsonism (14 cases), seizures (20 cases), psychiatric symptoms (9 cases), hypoacusia (6 cases), hydrocephalus (5 cases), stroke (1 case).

44 patients were diagnosed with GD type I, 1 patient with GD type II, 79 patients with GD type III. From 108 patients with available genotype, 2 were N370S/N370S, 2 N370S/L444P, 57 L444P/L444P, 3 N370S/Other variant, 18 L444P/Other variant, 5 D409H/D409H, 1 G377S/G377S, 23 with other variants.

### Peripheral neuropathy

77 patients from 9 studies evaluating 145 adults GD patients with neurological symptoms were diagnosed with neuropathy, encompassing 50 clinical manifest polyneuropathy, 16 asymptomatic polyneuropathy diagnosed by neurophysiology, and 11 cases of carpal tunnel syndrome (Table 1). Neurophysiology was available for 56 out of 66 patients with polyneuropathy, resulting in 35 cases of axonal neuropathy (12 sensory, 17 motor, 6 mixed) and 2 cases of mild demyelinating motor polyneuropathy, while 19 cases from one single study presented normal nerve conduction studies but abnormal quantitative sensory testing and epidermal denervation at skin biopsy. We excluded cases reporting only subjective symptom of paresthesia, without objective clinical or neurophysiological assessment.

14.3% of patients (n = 11/77 patients) presented peripheral neuropathy associated with other neurological symptoms, encompassing Parkinsonism (6 cases), cerebellar symptoms (1 case), myoclonus (1 case), oculomotor abnormalities (2 cases), stroke (2 cases), migraine (2 cases).

77 patients were diagnosed with GD type I and only one patient with GD type III. From 48 patients with available genotype, 8 were N370S/N370S, 10 N370S/L444P, 20 N370S/Other variant, 2 L444P/Other variant, 8 with other variants. No patients with L444P/L444P genotype were reported.

### Cognitive dysfunction

87 patients from 31 studies evaluating 366 adults GD patients with neurological symptoms manifested cognitive dysfunction, encompassing mild cognitive impairment (MCI), dementia, and lack of concentration (Table 1).

63 of 87 patients presented cognitive dysfunction in the context of a Parkinsonism (including 11 cases of LBD, see Sect. “Parkinsonism”).

80 patients were diagnosed with GD type I and 7 patients with GD type III. From 48 patients with available genotype, 20 were N370S/N370S, 7 N370S/L444P, 15 N370S/Other variant, 1 L444P/Other variant, 1 G377S/G377S, 6 with other variants. No patients with L444P/L444P genotype were reported.

### Psychiatric symptoms

63 patients from 12 studies evaluating 138 adults GD patients with neurological symptoms manifested psychiatric symptoms, described as depression, psychosis, anxiety, neurosis, irritability, hallucinations (Table 1).

51 of 63 patients presented psychiatric symptoms in the context of a Parkinsonism (see Sect. “Parkinsonism”)

53 patients were diagnosed with GD type I, 1 patient with GD type II, and 9 patients with GD type III. From 34 patients with available genotype, 9 were N370S/N370S, 4 N370S/L444P, 8 L444P/L444P, 4 N370S/Other variant, 4 L444P/Other variants, 8 with other variants.

### Seizures

48 patients from 23 studies evaluating 192 adults GD patients with neurological symptoms presented seizures during their disease course (Table 1). 13 patients presented this symptom in the context of myoclonic epilepsy (see Sect. “Myoclonus”). Of the other cases in which type of seizures was reported, 1 was described as absence, 3 as generalized tonic/clonic, 4 as generalized (without other description), 2 as partial seizures.

10 patients were diagnosed with GD type I and 38 patients with GD type III. From 32 patients with available genotype, 11 were L444/L444P, 1 N370S/L444P, 1 N370S/Other variant, 3 L444P/Other variants, 17 with other variants, and none with the N370S/N370S genotype.

### Myoclonus

43 patients from 18 studies evaluating 175 adult GD patients with neurological symptoms manifested myoclonus during their disease course (Table 1). 13 patients presented this symptom in the context of myoclonic epilepsy (see Sect. “Seizures”). Beyond a case with a description of myoclonic jerks, there was no other in-depth description of the location or magnitude of the myoclonic movements.

11 patients were diagnosed with GD type I and 32 patients with GD type III. From 34 patients with available genotype, 11 were L444/L444P, 1 N370S/Other variant, 6 L444P/Other variants, 1 G377S/ G377S, 15 with other variants, and none with the N370S/N370S genotype.

### Cerebellar symptoms

Cerebellar symptoms were found in 19 patients from 14 studies evaluating 84 adults GD patients with neurological symptoms (Table 1). All patients described in detail were reported to have an ataxic gait, and one case was described as having limb ataxia, in the absence further characterization. MRI data were available for 4 of these patients, of which 2 described mild atrophy, 1 minimal ischemic lesions, and only in 1 case there was a description of cerebellar and brainstem atrophy. Two case reports reported a follow-up over time, one case described ataxia started at 18 years old and worsened after 5 years, but with improvement one year after starting combined ERT therapy (miglustat/imiglucerase) [21], and one case (described as type II) presented worsening of cerebellar syndrome started from age of 4 years until the last evaluation at 26 years of age [22].

4 patients were diagnosed with GD type I, 1 patient with GD type II and 14 patients with GD type III. From 12 patients with available genotype, 5 were L444/L444P, 2 N370S/Other variant, 3 L444P/Other variants, 2 with other variants, and none with the N370S/N370S genotype.

### Other symptoms

The other neurological symptoms reported with a lower frequency, and/or not classifiable in the previous paragraphs, were:

- Non parkinsonian-tremors, described in 30 patients from 8 studies evaluating 140 adults GD patients with neurological symptoms.
- Hypoacusia, described in 22 patients from 8 studies evaluating 134 adults GD patients with neurological symptoms.
- Mental delay, described in 24 patients from 10 studies evaluating 458 adults GD patients with neurological symptoms.
- Neurological complications from bone disease, described in 14 patients from 9 studies evaluating 68 GD patients with neurological symptoms. These complications encompassed spinal cord compression and compressive neuropathy.
- Dystonia (2 patients from 2 studies).
- Hydrocephalus (5 patients from 3 studies).
- Spastic paraplegia (3 patients from 1 study).
- Stroke (3 patients from 2 studies).
- Single nerve palsy due to cerebral artery aneurism (1 patient from a case report).

### Genotype characterization

Genotype characterization was available for 388 GD patients from 62 studies (Supplementary file 3).

85 patients were N370S/N370S, 64 were L444P/L444P, 49 patients were N370S/L444P, 5 were D409H/D409H, and 3 were G377S/G377S. 110 patients presented the N370S associated with other variants, 30 patients the L444P associated with other variants, and 42 patients presented other combined variants.

94.1% (n = 80/85) N370S/N370S patients presented with Parkinsonism. No patients with this genotype presented with seizure, myoclonus, or cerebellar symptoms. Only 2 patients (2.4%) presented with oculomotor abnormalities.

89.1% (n = 57/64) L444P/L444P patients presented with oculomotor abnormalities, 14 with mental delay (21.9%). 2 patients (3.2%) presented with Parkinsonism, and no patients with neuropathy.

73.5% (n = 36/49) N370S/L444P patients presented with Parkinsonism, and 10 patients (20.4%) with neuropathy. No patients presented cerebellar symptoms, myoclonus or epilepsy, and only 2 (4.1%) presented with oculomotor abnormalities.

Of the 110 patients presenting other variants combined with N370S, the most frequent were: 9 N370S/V394L, 8 N370S/IVS2 + 1, 8 N370S/84GG, 6 N370S/RecNciL, 5 N370S/c.84insG, 4 N370S/R496H, 3 N370S/370Rec, 2 N370S/c.500insT, 2 N370S/c.1263-1317del, 2 N370S/IVS4-2A.G;(-203)A.G. 77 patients presented with Parkinsonism (70%) and 20 with neuropathy (18.2%). 3 patients (2.7%) presented with oculomotor abnormalities, 2 (1.8%) with cerebellar symptoms (in the context of a Parkinsonism), 1 (0.9%) with myoclonus and 1 (0.9%) with seizures.

Of the 30 patients presenting other variants combined with L444P, the most frequent were: 8 L444P/D409H, 7 L444P/R463C. In 18 of these patients (60%) oculomotor abnormalities were reported, and in 8 patients (26.7%) Parkinsonism.

Of the 42 patients presenting other variants, the most frequent were: 5 R463C/RecNciL, 3 C5390G/ C5390G, 2 G377S/Y205C, 2 R463C/R120W, 2 1342C/1448C.

## Discussion

The results of our systematic review reveal a broad spectrum of neurological symptoms in adult GD patients, extending beyond the traditionally defined neuronopathic types. We found that from 85 studies reporting data on 4190 patients with GD, more than 13% presented with at least one neurological symptom. The data are obtained mainly from subjects with type I GD, which is not surprising given the longer survival of these patients; however, it is worth considering that, according to the definition of GD types, type I patients should not present neurological manifestations [1, 17, 23]. A relatively large number of patients with GD type III exhibited neurological symptoms in adulthood, but only in a limited percentage these symptoms had an onset in adulthood. Only one type II patient was described, with an onset of neurological symptoms at 6 months, but with a clinical history not typical for the classic description of this type of GD, considering its survival with progressive neurological deterioration until the age of 26 [22].

Parkinsonism was the most frequently reported neurological issue in adulthood, mainly characterized by features consistent with idiopathic PD, and in a few cases with LBD, with a median age at onset of 51.9 years, lower than the typical age of PD onset in the general population [24]. As a valuable element, some PD patients also received the diagnosis confirmation in post-mortem analysis [25, 26]. The high report of PD in GD patients is not surprising, considering the relatively recent evidence that subjects carrying a heterozygous variant of the *GBA1* gene are at higher risk of developing PD [2, 4, 27]. No clear differences were found in the age at onset of PD between *GBA1* variant carriers reported in the literature (from 52.4 and 53.5 years in two papers from Italian groups and 57.2 from the paper of Gan-Or et al. of 2008) compared to the findings of our study in GD patients [27–29]. Moreover, patients with GD-related parkinsonism may present with a severe clinical picture, including poor responsiveness to levodopa, a higher presence of invalidating axial symptoms, cognitive impairment, and psychiatric issues.

Interestingly, all patients with peripheral neuropathy were classified as type I GD, possibly suggesting that this neurological complication is typical of patients with a mild clinical phenotype, even in a subclinical form that should be monitored. The majority of patients presented with an axonal polyneuropathy, and a portion of the cases was asymptomatic, diagnosed only by means of electrophysiological evaluation.

Oculomotor abnormalities were frequently reported in adult GD patients; however, this problem is only rarely found as an isolated neurological sign, and it is often part of complex syndromes, predominantly in GD type III patients, and less frequently in GD type I in the context of Parkinsonism. From a clinical perspective, a useful insight could be the frequent observation of slowed horizontal saccades, represented in both the mildest and most severe forms of the disease.

In the vast majority of cases, seizures, myoclonus, cerebellar symptoms, and other less common neurological manifestations were not found as isolated symptoms, but as part of complex neurological syndromes: this observation further underlines the complexity of GD-related neurological involvement. Moreover, the description of these symptoms in most cases was scarcely detailed, thus preventing their in-depth analysis. However, it can still be noted that the age at onset of these symptoms appears to be lower than others, as well as the aforementioned oculomotor disorders.

Complete information on genotype was not available for all patients; however, consistent data were achieved for the most frequent genotypes, and support previous evidence [30, 31]. Considering the huge variety of genotypes found, we focused on the most frequently reported. The N370S/N370S genotype was the most commonly reported in patients with Parkinsonism, with 80 cases described (26.9% of Parkinsonism found). 113 cases were described with heterozygous N370S variant associated with a second different variant (38.1%), and 8 with a homozygous or heterozygous L444P variant associated with a second different variant (2.7%). Among the cases with more severe neurological symptoms, as epilepsy, myoclonus, cerebellar symptoms or mental delay, the most frequently observed variant was the L444P, both in homozygosis or associated with other rare variants. Oculomotor abnormalities were not described with the N370S/N370S genotype, except in two cases associated with Parkinsonism [32, 33]; these disturbances were frequently reported with the L444P variant, especially in homozygosis. Accordingly, the data emphasizes the need for further insight into the genotype–phenotype correlations and the pathophysiological mechanisms underlying these neurological symptoms [12, 14, 23, 34, 35].

One of the critical challenges highlighted in our review is the potential delay in the diagnosis of GD, especially in cases with mild or nonspecific systemic symptoms. The presence of adult GD patients with new and unexplained neurological symptoms—in particular parkinsonism, neuropathy, epilepsy, neuropsychological issues, or oculomotor abnormalities—coupled with mild organomegaly, abnormal blood tests (as anemia, leukopenia and thrombocytopenia), or bone fragility, should lead to the consideration of GD as a potential diagnosis in the differential assessment. Early diagnosis is crucial for initiating appropriate treatment and preventing disease progression [36, 37]. Unfortunately, available existing therapies such as enzyme replacement therapy (ERT) and substrate reduction therapy (SRT) are not able to cross the blood brain barrier and therefore not suitable for the treatment of neurological complications of GD [1], although there was a description of neurological improvement from combined a single case report treated with combined ERT [21].

The classification of GD subtypes of our review is based on the report of the articles included in the systematic review analysis; however, it is important to take into account that some works reclassified type I patients as type III after some years on the basis of the new appearance of neurological symptoms. Moreover, the evaluation of a patient classified as type II in one case report could be subject to reconsideration, as it has been repeatedly reported that such patient should not have survived beyond the first years of life [22]. Therefore, as already suggested by previous work, our findings highlight certain limitations of the conventional classification of GD: the distinction into various GD subtypes based on the presence or absence of neurological manifestations should be reconsidered—at least in adults—considering that type I can frequently present with specific neurological phenotypes [12, 16, 38]. Further studies, potentially considering also neuroimaging data, which are still scarce in the literature, could contribute to understand which subjects are more at risk of developing a phenotype with neurological manifestations.

We acknowledge some important limitations in our study. First, the heterogeneity of the available studies, with variability in study design, different sample sizes and diagnostic criteria, and incomplete data particularly regarding genotypes, may introduce potential biases into our results. Second, the lack of prospective studies specifically focused on GD and its neurological aspects makes it challenging to establish causal relationships between genotypes and phenotypes, and to determine the trajectory and natural history of neurological symptoms in these patients. Third, the majority of the reviewed studies involved cohorts of patients with symptoms onset from childhood, which may have influenced the observed clinical picture. Nevertheless, it is essential to acknowledge the potential for missed diagnoses until adulthood, especially if systemic symptoms are mild.

In conclusion, to the best of our knowledge, this is the first systematic review evaluating the broad spectrum of the neurological manifestations in adult GD patients. The frequent involvement of the nervous system in adult GD challenges the conventional classification of GD types: clinicians should maintain a high level of suspicion for neurological involvement in all GD patients, even in those formerly categorized as type I GD, and consider a comprehensive neurological evaluation as part of their clinical assessment. The presence of Parkinsonism, often classic PD or even LBD, is common in GD adult patients and this should be considered. Further research is required to elucidate the underlying mechanisms of neurological symptoms in GD and develop targeted interventions for affected individuals.

### Supplementary Information

Below is the link to the electronic supplementary material.

Supplementary file1 (DOCX 17 KB)

Supplementary file2 (DOCX 13 KB)

Supplementary file3 (DOCX 56 KB)

## Data Availability

The data that support the findings of this study are available in anonymized dataset from the corresponding author, upon reasonable request.
