# Supplementary material for: Neurological symptoms in adults with Gaucher disease: a systematic review
Source: J Neurol. 2024 May 21;271(7):3897–907. doi: 10.1007/s00415-024-12439-5 (PMC11233309; doi:10.1007/s00415-024-12439-5)
Supplement: Supplementary file 2 — Supplementary file2 (DOCX 13 KB) [file 415_2024_12439_MOESM2_ESM.docx]

**Supplementary file 2. Search strategies**

Relevant articles were identified using the following keywords: (Gaucher disease [MeSH Term] OR GBA OR Glucosylceramidase [MeSH Term]) AND (Nervous System Diseases [MeSH Term] OR cognitive dysfunction [MeSH Term] OR dementia [MeSH Term] OR neuropathy OR Parkinson disease [MeSH Term] OR Parkinsonian Disorders [MeSH Term] OR ataxia [MeSH Term] OR epilepsy [MeSH Term] OR polyneuropathies [MeSH Term]).

The article types search included: Case Reports, Classical Article, Clinical Study, Clinical Trial, Comment, Comparative Study, Corrected and Republished Article, Electronic Supplementary Materials, Lecture, Letter, Multicenter Study, Observational Study, Preprint, Published Erratum, Research Support U.S. Gov't, Research Support Non-U.S. Gov't, Technical Report, Twin Study, Validation Study. A filter for age (“adult: 18+ years”) and for species (“Humans”) was also applied.

No restrictions were applied to sex, disease duration, disease severity, or follow-up duration.
