# Supplementary material for: Neurological symptoms in adults with Gaucher disease: a systematic review
Source: J Neurol. 2024 May 21;271(7):3897–907. doi: 10.1007/s00415-024-12439-5 (PMC11233309; doi:10.1007/s00415-024-12439-5)
Supplement: Supplementary file 3 — Supplementary file3 (DOCX 56 KB) [file 415_2024_12439_MOESM3_ESM.docx]

**Supplementary File 3. Description of the included studies, demographic data of participants, and full reference list of the 85 studies.**

.

| **Study ID/Study design** | **No. GD**  **No. GD with neurological symptoms (M-F)** | **Gaucher type** | **GBA mutation (extended)** | **Age at evaluation**  **Age at onset of neurological symptoms**  **Age at onset of GD** | **Type of neurological symptoms (number of patients)** | **Quality assessment** |
| --- | --- | --- | --- | --- | --- | --- |
| Racki 2021  Case Report | 1  1 (M) | 1 | H294Q, N370S, D409H (compound heterozygosis) | 43  34  34 | Parkinsonism | NA |
| Reynolds 2020 Case Report | 1  1 (F) | 1 | N370S/N370S | 69  69  68 | Left sixth nerve palsy due to inferior cerebellar artery aneurysm | NA |
| Le Peillet 2018 Case Report | 1  1 (M) | 1 | N370S/N370S | 62  44  NR | Parkinsonism (PD) with cognitive dysfunction | NA |
| Keshavaraj 2018 Case Report | 1  1 (M) | 1 | NR | 61  61  NR | Parkinsonism (PD) with Cognitive dysfunction and Psychiatric symptoms; Seizures | NA |
| Singh 2017  Case Report | 1  1 (M) | 3 | NR | 32  17  NR | Myoclonic epilepsy; Cognitive dysfunction (gaze palsy); Cerebellar symptoms; Oculomotor dysfunction | NA |
| Sawicka-Gutaj 2016 Case Report | 1  1 (M) | 3 | L444P/L444P | 26  24  1 | Oculomotor dysfunction (limited abduction of both eyes) | NA |
| Rim 2016 Case Report | 1  1 (F) | 3 | N188S/R257Q | 31  23  23 | Myoclonic epilepsy | NA |
| Lebouvier 2014  Case Report | 1  1 (F) | 1 | NR | 50  49  32 | Parkinsonism (PD) | NA |
| Diamandis 2014 Case Report | 1  1 (M) | 1 | NR | 79  78  NR | Parkinsonism (PD) with cognitive dysfunction; Neurological complications of bone disease | NA |
| Chauhan 2013 Case Report | 1  1 (M) | 3 | R463C/RecNcil | 36  24  NR | Cognitive dysfunction; Seizures; Oculomotor dysfunction (horizontal gaze palsy) | NA |
| Aksu 2011  Case Report | 1  1 (F) | 3 | NR | 20  20  10 | Oculomotor dysfunction (apraxia) | NA |
| Alonso-Canovas 2010 Case Report | 1  1 (M) | 1 | N370S/L444P | 66  60  17 | Parkinsonism (atypical) with cognitive and oculomotor dysfunction (supranuclear gaze palsy) | NA |
| Colak 2009  Case Report | 1  1 (F) | 1 | NR | 38  35  23 | Neurological complications of bone disease | NA |
| Capablo 2007 | 1  1 (M) | 1 | L444P/E326K+N188S | 31  22  5 | Myoclonic epilepsy; Cerebellar symptoms; | NA |
| Itokawa 2006  Case Report | 1  1 (M) | 1 | L444P/R463C | 42  42  31 | Parkinsonism (PD) | NA |
| Hughes 2006  Case report | 1  1 (M) | 1 | N370S/N370S | 74  74  67 | Parkinsonism (atypical) | NA |
| Spitz 2006 Case Report | 1  1 (M) | 1 | N370S/L444P | 45  43  22 | Parkinsonism (PD); Neurological complications of bone disease | NA |
| Hamlat 2004  Case Report | 1  1 (M) | 1 | N307S/1263del 55 | 65  55  55 | Parkinsonism (PD); Seizures | NA |
| Guimarães  2003 Case Report | 1  1 (M) | 1 | G377S/G377S | 53  49  46 | Parkinsonism (atypical) with cognitive and oculomotor dysfunction (supranuclear gaze palsy); Myoclonus; | NA |
| Várkonyi 2002  Case Report | 1  1 (M) | 1 | N370S/V394L | 63  63  62 | Parkinsonism (atypical) with Cerebellar symptoms | NA |
| Tayebi 2001 Case Report | 1  1 (F) | 1 | L444P/D409H | 48  42  19 | Parkinsonism (atypical) with Myoclonus and Oculomotor dysfunction (slowed horizontal saccades) | NA |
| Aoki 2001 Case Report | 1  1 (M) | 1 | L444P/L444P | 20  19  1 | Oculomotor dysfunction | NA |
| Baumann 2001 Case Report | 1  1 (M) | 2 | L444P/D409H | 26  1  1 | Cerebellar and Pyramidal syndrome with Oculomotor dysfunction (limited horizontal eye movements), Dystonia and Psychiatric symptoms | NA |
| Tuzun 2000 Case Report | 1  1 (M) | 3 | NR | 27  15  14 | Seizures | NA |
| Machaczka 1999 Case Report | 1  1 (M) | 1 | N370S/IVS2+1 | 51  39  39 | Parkinsonism (PD) | NA |
| Seeman 1996  Case Report | 1  1 (M) | 3 | V398L/T491I | 22  NR  NR | Myoclonic epilepsy; Mental delay | NA |
| McAlarney 1995 Case Report | 1  1 (F) | 1 | NR | 53  44  NR | Cerebellar symptoms; Neuropathy; Oculomotor dysfunction (left sixth nerve palsy, resolved); Right optic neuritis | NA |
| Goldblatt 1987 Case Report | 1  1 (M) | 1 | NR | 58  NR  NR | Neurological complications of bone disease | NA |
| Seehra 2020 Case Series | 5  3 (1-2) | 3 | 3 L444P/L444P | 20 (Range 18–22)  8,5  1,5 | Cerebellar symptoms (3); Mental delay (3); Seizures (1); Oculomotor dysfunction (3, slowed horizontal saccades); Tremor (2); Hypoacusia (1) | Fair |
| Andréasson 2019  Case Series | 19  2 (1-1) | 1 (n=2) | 1 L444P/L444P  1 L444P/G241A | 68,5 (Range 64–73)  NR  57 | Mild motor demyelinating Neuropathy (2) | Fair |
| Kurolap 2019  Case Series | 4  3 (2-1) | 3 | 3 D409H/D409H | 20,1 (1,8)  4,7 (1,2)  3 (1,7) | Mental delay (1); Oculomotor dysfunction (3; 1 opsoclonus, 2 apraxia) | Fair |
| Collins 2018 Case Series | 5  5 (1-4) | 3 | 3 N370S/L444P  1 L444P/R463C  1 R463C/R463C | 59 (8)  54,4 (10)  36 (8,4) | Parkinsonism (5 PD) with Cognitive dysfunction (2) | Fair |
| Rodriguez-Porcel 2017  Case Series | 2  2 (2-0) | 2 | 1 N370S/N370S  1 N370S/L444P | 45 (3)  43 (2)  23 (21.5) | Parkinsonism (2 PD) | Good |
| Sestito 2017 Case Series | 5  4 (3-1) | 3 | 4 L444P/L444P | 37 (9)  13 (7)  2 (1) | Myoclonus (3); Cerebellar symptoms (1); Mental delay (4); Myoclonic epilepsy (1); Oculomotor dysfunction (3 supranuclear gaze palsy); Psychiatric symptoms (4) | Good |
| Detollenaere 2017  Case Series | 4  3 (NA) | 1 | 1 L444P/RECTL  1 D409H/D409H  1 D409H/L444P | 23,8 (3,9)  9,9 (9)  9,9 (9) | Myoclonic epilepsy (1); Cerebellar symptoms (1); Mental delay (2); Oculomotor dysfunction (2 ophtalmoplegia) | Fair |
| Lopez 2016 Case Series | 19  19 (10-9) | 1 (n=18)  3 (n=1) | 9 N370S/N370S  2 N370S/L444P  2 N370S/c.84insG  1 N370S/IVS2+1  1 N370S/rec  1 N370S/R257Q  1 N370S/55bpdel  1 N370S/V394L  1 L444P/D409H | NR  49,7 (8,9)  27,6 (16) | Parkinsonism (18: 4 LBD, 14 PD) with Psychiatric symptoms (13) and Cognitive dysfunction (2 other than DLB); Functional tremor (1) | Good |
| Monestime 2016  Case Series | 5  5 (1-4) | 1 (n=4)  3 (n=1) | 1 N370S/N370S  1 N370S/L444P  2 N370S/c.84insG  1 D409H/L444P | NR  51,8 (8,7)  21 | Parkinsonism (5: 3 DLB, 2 PD), 1 with oculomotor dysfunction (slowed horizontal saccades) | Good |
| Ben Rhouma 2012  Case Series | 3  1 (M) | 1 | N370S/RecNciI | 52  52  46 | Parkinsonism with Cerebellar dysfunction (1); Neurological complications of bone disease (1) | Good |
| Saunders-Pullman 2010 Case Series | 4  4 (3-1) | 1 | 2 N370S/N370S  2 N370S/R496H | 60,5 (5,4)  56,5 (7)  56,5 (7) | Parkinsonism (4: 1 PD, 2 PD with cognitive dysfunction, 1 atypical with cognitive dysfunction) | Good |
| Kraoua 2009 Case Series | 10  10 (6-4) | 1 | 1 N370S/N370S  2 N370S/L444P  1 N370S/RecNciI | 65,8 (10)  56,3 (9,3)  41 (20,4) | Parkinsonism (10: 3 DLB, 7 PD) with Cognitive dysfunction (3, other than DLB) | Good |
| Tajima 2009 Case Series | 42  12 (7-5) | 1 (n=5)  3 (n=7) | 6 L444P/L444P  1 L444P/F123I  1 F213I/F213I  1 F213I/D409H  1 D409H/IVS10-1  1 D409H/D409H  1 N188S/55bpdel | 34,5 (12,3)  24,5  13,8 (14,8) | Seizures (6); Myoclonus (3); Mental delay (3); Oculomotor dysfunction (8; 4 apraxia, 2 strabismus, 2 NR); Cerebellar symptoms (1); Tremor (1); Hypoacusia (1); Hydrocephalus (1) | Good |
| Goker-Alpan 2008  Case Series | 7  7 (5-2) | 1 | 4 N370S/N370S  1 N370S/L444P  1 N370S/c.84dupG  1 N370S/RecNcil | NR  50,7 (8,3)  32,1 (17,4) | Parkinsonism (7 PD) with Cognitive dysfunction (3) | Good |
| Kono 2007  Case Series | 1  1 (M) | 3 | NR | 38  7  6 | Parkinsonism (PD) with Seizures and Oculomotor dysfunction (slowed horizontal saccades) | Fair |
| Raja 2007  Case Series | 2  2 (M) | 1 | 2 L444P/L444P | 50,5 (2,12)  45 (5,66)  45 (5,66) | Parkinsonism (2 atypical) with Cognitive dysfunction (2), Oculomotor dysfunction (2, supranuclear gaze palsy), Psychiatric symptoms (2) and Dystonia (1) | Good |
| Capablo 2007 Case Series | 31  11 (2-9) | 1 (n=10)  3 (n=1) | 4 N370S/L444P  2 N370S/ IVS4-2A.G;(-203)A.G  1 N370S/L336P  1 N370S/c.500insT  1 L444P/D409H  1 N370S/G195W  1 N370S/V398I | 51,2 (15,9)  NR  NR | Neuropathy (11); Parkinsonism (2 PD, 1 indefinite) with Cognitive dysfunction (1); Myoclonus (2); Oculomotor dysfunction (1, NR); Hypoacusia (3); Stroke (2); Migraine (3) | Good |
| Bembi 2003 Case Series | 58  4 (1-3) | 1 | 2 N370S/NR  1 G377S/G377S  1 N370S/L444P | 50,2 (8)  50,2 (8)  41 (20,4) | Parkinsonism (4 PD); Hypoacusia (4) | Fair |
| Várkonyi 2003 Case Series | 4  4 (3-1) | 1 | 1 N370S/N370S  1 N370S/V394L  1 N370S/ c.1263-1317del  1 R463C/R120W | 54,5 (9,2)  46,7 (9,7)  33,7 (26,7) | Parkinsonism (3 PD, 1 indefinite) with Oculomotor dysfunction (2; 1 slowed horizontal saccades, 1 NR) | Good |
| Park 2003  Case Series | 16  8 (4-4) | 3 | 1 L444P/L444P  1 K157Q/D140H+ E326K  1 N188S/RecNciI (Fusion)  1 N188S/c.84-85insG  1 N188S/Recomb (Fusion)  1 G377S/Y205C  1 N188S/RecNcil  1 c.1263–1317del/ F216y | 28,4 (5,7)  14,4 (5,6)  8,7 (5,6) | Myoclonic epilepsy (5); Seizures (3); Myoclonus (2); Mental delay (2); Cerebellar symptoms (2) | Fair |
| Poll 2000  Case Series | 4  1 (M) | 1 | L444P/L444P | 47  47  18 | Neurological complications of bone disease (1) | Fair |
| Parenti 1998 Case Series | 3  3 (F) | 3 | 3 C5390G/C5390G | 26 (2,1)  18,7 (3,5)  15,7 (3,5) | Seizures (3) | Fair |
| Beutler 1995 Case Series | 8  2 (F) | 3 | 1 1342C/1342C  1 1342C/1448C | 28,5 (14,8)  NR  NR | Oculomotor dysfunction (2, apraxia); Hydrocephalus (1) | Poor |
| Balicki 1995 Case Series | 5  1 (F) | 3 | 1 1342C/1448C | 38  NR  NR | Oculomotor dysfunction (1, supranuclear gaze palsy) | Fair |
| Uyama 1992 Case Series | 3  3 (2-1) | 3 | NR | 34,6 (7,5)  10,6  NR | Oculomotor dysfunction (3; 2 slowed horizontal saccades, 1 supranuclear gaze palsy); Myoclonus (1); Hydrocephalus (3) Spastic paraplegia (3); Cerebellar symptoms (3); Cognitive dysfunction (2); Mental delay (1) | Good |
| Hermann 1989 Case Series | 3  3 (2-1) | 1 | NR | 55,3 (21,8)  55,3 (21,8)  36,7 | Neurological complications of bone disease (3) | Fair |
| D'amore 2021 Cohort study | 250  45 (NA) | 1 (n=18)  3 (n=27) | 20 L444P/L444P  5 L444P/R463C  4 R463C/RecNcil  2 L444P/D409H  1 L444P/P266R  1 L444P/P266A  1 L444P/E233D  1 R463C/IVS2+1  1 R463C/G377R  1 R463C/R257Q  1 R463C/R496C  1 R262G/RecNcil  1 H311R/R359Q  1 R463C/D409H  1 L240V/L240V  1 R359Q/H311R  2 NR | NR  NR  NR | Oculomotor dysfunction (42; 27 slowed horizontal saccades, 15 abnormal saccades at video-oculography)  Intentional tremor (14)  Parkinsonism (7 PD)  Seizures (6)  Myoclonus (5)  Cognitive dysfunction (2)  Cerebellar symptoms (1) | Fair |
| Lopez 2019 Cohort study | 18  9 (6-3) | 1 | 3 N370S/N370S  2 N370S/L444P  1 N370S/IVS2+1  1 N370S/c.203delC  1 N370S/V394L  1 N370S/c.84insG | 61 (5,6)  52,1 (8,4)  25,5 (21,1) | Parkinsonism (1 DLB, 8 PD) with Psychiatric symptoms (7) and cognitive dysfunction (1 other than DLB) | Good |
| Steward 2019 Cohort study | 34  5 (F) | 3 | 4 L444P/L444P  1 G377S/Y205C | 25 (5,4)  1,7 (1,6)  1,7 (1,6) | Seizures (4); Myoclonus (1); Oculomotor dysfunction (2, NR) | Good |
| Thaler 2017 Cohort study | 12  12 (8-4) | 1 | 3 N370S/N370S  3 N370S/370Rec  2 N370S/R496H  2 N370S/V394L  1 N370S/ IVS2+1  1 N370S/L444P | 58.4 (10.5)  51,9 (10,7)  NR | Parkinsonism (12 PD) with cognitive dysfunction (5) | Fair |
| Devigili 2017 Cohort study | 25  19 (9-10) | 1 | 4 N370S/L444P  3 N370S/N370S  2 N370S/RecNcil  2 N370S/ g.4179_5042conJ03060.  1:g.2367_2911  1 N370S/D399N  1 N370S/D409H  1 N370S/H255Q+D409H  1 N370S/W381C  1 N370S/g.−3091+834del3925  1 N370S/V214X  1 R48W/L444P  1 R170P /c.1225-10delC,c.1225-14 T > A | 41,4 (11,1)  NR  NR | Neuropathy (19) | Fair |
| Alcalay 2014 Cohort study | 427  11 (8-3) | 1 | N370S/N370S and N370S/NR (number of patients NR) | NR  54,2 (8,6)  54,2 (8,6) | Parkinsonism (11 PD) | Good |
| Kuter 2013 Cohort study | 115  3 (NA) | 3 | NR | NR  NR  NR | Aspecific tremor (3) | Fair |
| Kumar 2013 Cohort study | 2  2 (M) | 3 | 1 N370S/N370S  1 N370S/(D409H; H255Q) | 49,5 (9)  46 (7)  NR | Parkinsonism (2 PD) | Good |
| Chetrit 2013 Cohort study | 510  11 (9-2) | 1 | 5 N370S/N370S  2 N370S/V394L  1 N370S/L444P  1 N370S/IVS2+1  1 N370S/RecTL  1 N370S/84GG | 62,8 (1,1)  56,5 (10,9)  NR | Parkinsonism (11 PD) with Cognitive dysfunction (7) | Fair |
| Stirnemann 2012 Cohort study | 562  14 (NA) | 1 | 6 NR  1 N370S/N370S  5 N370S/L444P  2 N370S/OTHER | NR  NR  NR | Parkinsonism (14 PD) with Cognitive dysfunction (6) | Fair |
| Benko 2011 Cohort study | 15  5 (2-3) | 3 | 4 L444P/L444P  1 R463C/RecNciI+Rec7 | 21,4 (3,9)  2  2 | Oculomotor dysfunction (5, slowed horizontal saccades); Myoclonic epilepsy (1) | Fair |
| Rosenbloom 2011 Cohort study | 711  68 (38-30) | 1 | 27 N370S/N370S  9 N370S/L444P  13 N370S/NR  4 N370S/84GG  2 N370S/IVS2+1  2 L444P/NR  11 NR | 57 (10)  57 (10)  37 (20) | Parkinsonism (68 indefinite) with Cognitive dysfunction (16) | Fair |
| Giraldo 2011 Cohort study | 111  32 (NA) | 1 | NR | 40 (2–72)  26,7  26,7 | Neuropathy (10); Parkinsonism (8 PD); Hypoacusia (7); Cognitive dysfunction (5); Seizures (1); Oculomotor dysfunction (3; 2 strabismus, 1 slowed saccades); Psychiatric symptoms (2); Aspecific tremor (3); Stroke (1) | Fair |
| Lesage 2011 Cohort study | 2  2 (1-1) | 1 | 1 c.1263del+RecTL  1 N370S/RecΔ55 | 35  NR  NR | Parkinsonism (2) with Cognitive dysfunction (1) | Fair |
| Biegstraaten 2010 Cohort study | 103  17 (11-6) | 1 | 4 N370S/N370S  2 N370S/L444P  1 N370S/IVS2+1  10 N370S/NR | 61 (41–75)  NR  NR | Neuropathy (17) | Good |
| Chérin 2010 Cohort study | 105  51 (NA) | 1 | NR | 46 (14)  NR  22 (15) | Parkinsonism (26: 4 PD, 22 indefinite) with Cognitive dysfunction (1); Psychiatric symptoms (21); Neuropathy (11); Myoclonus (6); Seizures (2); Hypoacusia (2); Neurological complications of bone disease (1) | Fair |
| Bultron 2010 Cohort study | 444  11 (7-4) | 1 | 5 N370S/N370S  2 N370S/L444P  3 N370S/84GG  1 L444P/W312C | 55 (8,8)  55 (8,8)  29,1 (21,9) | Parkinsonism (11 PD) | Fair |
| Goker-Alpan 2008 Cohort study | 32  6 (2-4) | 3 | 5 L444P/L444P  1 G377S/Y305C | 22,2 (5,5)  13 (0,5)  13 (0,5) | Oculomotor dysfunction (6, slowed horizontal saccades); Mental delay (4); Seizures (2); Myoclonic epilepsy (1); Cognitive dysfunction (1) | Good |
| Mercimek-Mahmutoglu 2007 Cohort study | 9  2 (M) | 1 | 1 N370S/N370S  1 N370S/R120Q | 57,5 (6,4)  NR  NR | Neuropathy (2) | Good |
| Alfonso 2007 Cohort study | 193  16 (NA) | 1 | 5 N370S/L444P  1 N370S/N370S  9 N370S/Other  1 G377S/D409H | 28,5  NR  NR | Oculomotor dysfunction (6; 4 strabismus, 2 NR); Hypoacusia (3); Parkinsonism (3 indefinite) with Cognitive dysfunction (1); Essential tremor (3); Clonic movements (1) | Fair |
| Tylki-Szymañska 2006 Cohort study | 47  7 (1-6) | 3 | 4 L444P/L444P  2 L444P/D409H  1 L444P/V305L | 33 (9)  6  NR | Oculomotor dysfunction (7, supranuclear gaze palsy); Psychiatric symptoms (3); Myoclonus (3) Cognitive dysfunction (1) | Poor |
| Clark 2005 Cohort study | 2  2 (NA) | 1 | 2 N370S/N370S | 72,5  72,5  NR | Parkinsonism (2 PD) with Cognitive dysfunction (1) | Good |
| Goker-Alpan 2004 Cohort study | 1  1 (F) | 1 | NR | 63  63  NR | Parkinsonism (PD) with Cognitive dysfunction | Fair |
| Aharon-Peretz 2004 Cohort study | 3  3 (NA) | 1 | 3 N370S/N370S | 60 (14,2)  60 (14,2)  NR | Parkinsonism (3 PD) | Good |
| Pastores 2003 Cohort study | 55  10 (NA) | 1 | NR | 46,8  NR  NR | Neuropathy (3); Parkinsonism (3 PD); Tremor (4) | Poor |
| Tayebi 2003 Cohort study | 17  17 (12-5) | 1 | 5 N370S/N370S  1 N370S/L444P  1 N370S/V394L  1 N370S/IVS2+1  1 N370S/c.84-85insG  1 N370S/c.1263-1317del  1 N370S/c.500insT  1 N370S/RecNciL  2 N370S/NR  1 L444P/D409H+duplication  1 R463C/R120W  1 G377S/G377S | 47,9 (8,1)  47,9 (8,1)  36,3 | Parkinsonism (17 PD) with Cognitive dysfunction (6); Oculomotor dysfunction (2, slowed horizontal saccades) | Fair |
| Ida 1999 Cohort study | 15  5 (3-2) | 1 | 5 L444P/L444P | 29,2 (6,4)  6,6  2,8 (1,6) | Oculomotor dysfunction (5, supranuclear gaze palsy); Mental delay (3); Hypoacusia (2); Psychiatric symptoms (1); Seizures (1) | Fair |
| Rice 1996 Cohort study | 35  1 (NA) | 3 | NR | 18  18  18 | Psychiatric symptoms (1); Seizures (1); Cerebellar symptoms (1) | Poor |
| Neudorfer 1996 Cohort study | 6  6 (3-3) | 1 | NR | 48,8  48,8  NA | Parkinsonism (6 PD) with Psychiatric symptoms (2) and Myoclonus (1) | Poor |
| Tylki-Szymańska 1996 Cohort study | 22  4 (2-2) | 1 (n=1)  3 (n=3) | 2 L444P/L444P  2 NR | 26,7(13,7)  NR  NR | Oculomotor dysfunction (4, apraxia); Parkinsonism (1 PD) | Poor |
| Grewal 1991 Cohort study | 8  8 (NA) | 1 | NR | 38 (15,6)  38 (15,6)  14,8 (8,3) | Neurological complications of bone disease (4) | Fair |

Age of patients is reported as Mean (SD), calculated from the individual values if reported in the study.

**Legend**

GD: Gaucher Disease; GBA: Glucocerebrosidase; NR: Not reported; NA: Not applicable; PD: Parkinson’s Disease; LBD: Lewy Body Disease

**Reference list**

1. Racki V, Papic E, Almahariq F, et al (2021) The Successful Three-Year Outcome of Deep Brain Stimulation in Gaucher Disease Type 1 Associated Parkinson's Disease: A Case Report. Mov Disord Clin Pract 8:604–606. doi: 10.1002/mdc3.13285.
2. Reynolds MR, Heiferman DM, Boucher AB, et al (2020) Multiple Intracranial Aneurysms in a Patient with Type I Gaucher Disease: A Case Report and Literature Review. Br J Neurosurg 34:202–204. doi: 10.1080/02688697.2019.1691927.
3. Le Peillet D, Prendki V, Trombert V, et al (2018) Type I Gaucher Disease with Bullous Pemphigoid and Parkinson Disease: A Case Report. Medicine (Baltimore) 97:e0188. doi: 10.1097/MD.0000000000010188.
4. Keshavaraj A, Gajalakshan L (2018) Gaucher’s Disease Type I: A Case Report. Ceylon Med J 63:33–34. doi: 10.4038/cmj.v63i1.8640
5. Singh R, Choudhary A, Kumar AS, Goyal MK (2017) Progressive Myoclonic Epilepsy and Horizontal Gaze Palsy: A Rare Aetiology. BMJ Case Rep 2017:bcr2017222304. doi: 10.1136/bcr-2017-222304.
6. Sawicka-Gutaj N, Machaczka M, Kulińska-Niedziela I, et al (2016) The Appearance of Newly Identified Intraocular Lesions in Gaucher Disease Type 3 Despite Long-Term Glucocerebrosidase Replacement Therapy. Ups J Med Sci 121:192–195. doi: 10.1080/03009734.2016.1181196.
7. Rim JH, Baik M, Yoon SO, et al (2016) Clinical Utility of Bone Marrow Study in Gaucher Disease: A Case Report of Gaucher Disease Type 3 With Intractable Myoclonic Seizures. Ann Lab Med 36:177–179. doi: 10.3343/alm.2016.36.2.177.
8. Lebouvier T, Clairembault T, Devos D, et al (2014) Peripheral Autonomic Nervous System Involvement in Gaucher-Related Parkinsonism. J Parkinsons Dis 4:29–32. doi: 10.3233/JPD-130304.
9. Diamandis P, Amato D, Finkelstein J, Keith J (2014) 79-Year Old Man with Parkinsonism and Acute Spinal Cord Compression. Brain Pathol 24:101–102. doi: 10.1111/bpa.12090.
10. Chauhan V, Kumar RV, Mahesh DM, et al (2013) Adult Type 3 Gaucher Disease as Manifestation of R463C/Rec Nci I Mutation: First Reported Case in the World Literature. J Assoc Physicians India 61:346–348.
11. Aksu T, Baysal E, Bıyıkoğlu F, Tüfekçioğlu O (2011) Gaucher's Disease with Valvular, Myocardial and Aortic Involvement in a Patient with Oculomotor Apraxia. Anadolu Kardiyol Derg 11:E4–E5. doi: 10.5152/akd.2011.180.
12. Alonso-Canovas A, Katschnig P, Tucci A, et al (2010) Atypical Parkinsonism with Apraxia and Supranuclear Gaze Abnormalities in Type 1 Gaucher Disease: Expanding the Spectrum: Case Report and Literature Review. Mov Disord 25:1506–1509. doi: 10.1002/mds.23192.
13. Colak M, Canbaz H, Ayan I, et al (2009) Intrapelvic Mass Causing Femoral Compression Neuropathy in a Patient with Gaucher Disease: A Case Report. Eklem Hastalik Cerrahisi 20:169–173. doi: 10.5606/ehc.2009.036.
14. Capablo JL, Franco R, de Cabezón AS, et al (2007) Neurologic Improvement in a Type 3 Gaucher Disease Patient Treated with Imiglucerase/Miglustat Combination. Epilepsia 48:1406–1408. doi: 10.1111/j.1528-1167.2007.01135.x.
15. Itokawa K, Tamura N, Kawai N, et al (2006) Parkinsonism in Type I Gaucher's Disease. Intern Med 45:1165–1167. doi: 10.2169/internalmedicine.45.1593.
16. Hughes DA, Ginsberg L, Baker R, et al (2007) Effective Treatment of an Elderly Patient with Gaucher's Disease and Parkinsonism: A Case Report of 24 Months' Oral Substrate Reduction Therapy with Miglustat. Parkinsonism Relat Disord 13:365–368. doi: 10.1016/j.parkreldis.2006.07.010.
17. Spitz M, Rozenberg R, Silveira PA, Barbosa ER (2006) Parkinsonism in Type 1 Gaucher's Disease. J Neurol Neurosurg Psychiatry 77:709–710. doi: 10.1136/jnnp.2005.076166.
18. Hamlat A, Saikali S, Lakehal M, et al (2004) Cauda Equina Syndrome Due to an Intra-Dural Sacral Cyst in Type-1 Gaucher Disease. Eur Spine J 13:249–252. doi: 10.1007/s00586-003-0606-y.
19. Guimarães J, Amaral O, Sá Miranda MC (2003) Adult-Onset Neuronopathic Form of Gaucher's Disease: A Case Report. Parkinsonism Relat Disord 9:261–264. doi: 10.1016/S1353-8020(02)00085-9.
20. Várkonyi J, Simon Z, Soós K, Poros A (2002) Gaucher Disease Type I Complicated with Parkinson's Syndrome. Haematologia (Budap) 32:271–275. doi: 10.1163/156855902760275496.
21. Tayebi N, Callahan M, Madike V, et al (2001) Gaucher Disease and Parkinsonism: A Phenotypic and Genotypic Characterization. Mol Genet Metab 73:313–321. doi: 10.1006/mgme.2001.3216.
22. Aoki M, Takahashi Y, Miwa Y, et al (2001) Improvement of Neurological Symptoms by Enzyme Replacement Therapy for Gaucher Disease Type IIIb. Eur J Pediatr 160:63–64. doi: 10.1007/s004310000743.
23. Baumann N, Lefèvre M, Turpin JC, et al (2001) Atypical Course of Neuropathic Gaucher's Disease: Follow Up from Early Infancy Until Adulthood. J Neurol Neurosurg Psychiatry 70:133–134. doi: 10.1136/jnnp.70.1.133.
24. Tüzün E, Baykan B, Gürses C, Gökyigit A (2000) Longterm Follow-Up of Electroencephalographic and Clinical Findings of a Case with Gaucher's Disease Type 3a. Seizure 9:469–472. doi: 10.1053/seiz.2000.0457.
25. Machaczka M, Rucinska M, Skotnicki AB, Jurczak W (1999) Parkinson's Syndrome Preceding Clinical Manifestation of Gaucher's Disease. Am J Hematol 61:216–217. doi: 10.1002/(sici)1096-8652(199907)61:3<216::aid-ajh7>3.0.co;2-b.
26. Seeman PJ, Finckh U, Höppner J, et al (1996) Two New Missense Mutations in a Non-Jewish Caucasian Family with Type 3 Gaucher Disease. Neurology 46:1102–1107. doi: 10.1212/wnl.46.4.1102.
27. McAlarney T, Pastores GM, Hays AP, Latov N (1995) Antisulfatide Antibody and Neuropathy in a Patient with Gaucher's Disease. Neurology 45:1622–1623. doi: 10.1212/wnl.45.8.1622.
28. Goldblatt J, Keet P, Dall D (1987) Spinal Cord Decompression for Gaucher's Disease. Neurosurgery 21:227–230. doi: 10.1227/00006123-198708000-00015.
29. Seehra GK, Eghbali A, Sidransky E, FitzGibbon E (2020) White Vitreous Opacities in Five Patients with Gaucher Disease Type 3. Am J Med Genet A 182:808–812. doi: 10.1002/ajmg.a.61472.
30. Andréasson M, Solders G, Björkvall CK, et al (2019) Polyneuropathy in Gaucher Disease Type 1 and 3 - A Descriptive Case Series. Sci Rep 9:15358. doi: 10.1038/s41598-019-51874-y.
31. Kurolap A, Del Toro M, Spiegel R, et al (2019) Gaucher Disease Type 3c: New Patients with Unique Presentations and Review of the Literature. Mol Genet Metab 127:138–146. doi: 10.1016/j.ymgme.2019.05.016.
32. Collins LM, Williams-Gray CH, Morris E, et al (2018) The Motor and Cognitive Features of Parkinson's Disease in Patients with Concurrent Gaucher Disease Over 2 Years: A Case Series. J Neurol 265:1789–1794. doi: 10.1007/s00415-018-8924-4.
33. Rodriguez-Porcel F, Espay AJ, Carecchio M (2017) Parkinson Disease in Gaucher Disease. J Clin Mov Disord 4:7. doi: 10.1186/s40734-017-0050-3.
34. Sestito S, Filocamo M, Ceravolo F, et al (2017) Norrbottnian Clinical Variant of Gaucher Disease in Southern Italy. J Hum Genet 62:507–511. doi: 10.1038/jhg.2016.155.
35. Detollenaere C, Benghergbia M, Brassier A, et al (2017) Type 3 Gaucher Disease, Diagnostic in Adulthood. Mol Genet Metab Rep 13:1–2. doi: 10.1016/j.ymgmr.2017.05.001.
36. Lopez G, Kim J, Wiggs E, et al (2016) Clinical Course and Prognosis in Patients with Gaucher Disease and Parkinsonism. Neurol Genet 2:e57. doi: 10.1212/NXG.0000000000000057.
37. Monestime G, Borger DK, Kim J, et al (2016) Varied Autopsy Findings in Five Treated Patients with Gaucher Disease and Parkinsonism Include the Absence of Gaucher Cells. Mol Genet Metab 118:55–59. doi: 10.1016/j.ymgme.2016.04.007.
38. Ben Rhouma F, Kallel F, Kefi R, et al (2012) Adult Gaucher Disease in Southern Tunisia: Report of Three Cases. Diagn Pathol 7:4. doi: 10.1186/1746-1596-7-4.
39. Saunders-Pullman R, Hagenah J, Dhawan V, et al (2010) Gaucher Disease Ascertained through a Parkinson's Center: Imaging and Clinical Characterization. Mov Disord 25:1364–1372. doi: 10.1002/mds.23087.
40. Kraoua I, Stirnemann J, Ribeiro MJ, et al (2009) Parkinsonism in Gaucher's Disease Type 1: Ten New Cases and a Review of the Literature. Mov Disord 24:1524–1530. doi: 10.1002/mds.22680.
41. Tajima A, Yokoi T, Ariga M, et al (2009) Clinical and Genetic Study of Japanese Patients with Type 3 Gaucher Disease. Mol Genet Metab 97:272–277. doi: 10.1016/j.ymgme.2009.04.001.
42. Goker-Alpan O, Lopez G, Vithayathil J, et al (2008) The Spectrum of Parkinsonian Manifestations Associated with Glucocerebrosidase Mutations. Arch Neurol 65:1353–1357. doi: 10.1001/archneur.65.10.1353.
43. Kono S, Shirakawa K, Ouchi Y, et al (2007) Dopaminergic Neuronal Dysfunction Associated with Parkinsonism in Both a Gaucher Disease Patient and a Carrier. J Neurol Sci 252:181–184. doi: 10.1016/j.jns.2006.11.003.
44. Raja M, Azzoni A, Giona F (2007) Movement and Mood Disorder in Two Brothers with Gaucher Disease. Clin Genet 72:357–361. doi: 10.1111/j.1399-0004.2007.00872.x.
45. Capablo JL, Saenz de Cabezón A, Fraile J, et al (2008) Spanish Group on Gaucher Disease. Neurological Evaluation of Patients with Gaucher Disease Diagnosed as Type 1. J Neurol Neurosurg Psychiatry 79:219–222. doi: 10.1136/jnnp.2007.120121.
46. Bembi B, Zambito Marsala S, et al (2003) Gaucher's Disease with Parkinson's Disease: Clinical and Pathological Aspects. Neurology 61:99–101. doi: 10.1212/WNL.61.1.99.
47. Várkonyi J, Rosenbaum H, Baumann N, et al (2003) Gaucher Disease Associated with Parkinsonism: Four Further Case Reports. Am J Med Genet A 116A:348–351. doi: 10.1002/ajmg.a.10880.
48. Park JK, Orvisky E, Tayebi N, et al (2003) Myoclonic Epilepsy in Gaucher Disease: Genotype-Phenotype Insights from a Rare Patient Subgroup. Pediatr Res 53:387–395. doi: 10.1203/01.PDR.0000054292.09411.03.
49. Poll LW, Koch JA, vom Dahl S, et al (2000) Type I Gaucher Disease: Extraosseous Extension of Skeletal Disease. Skeletal Radiol 29:15–21. doi: 10.1007/s002560050588.
50. Parenti G, Filocamo M, Titomanlio L, et al (1998) A Novel Mutation of the Beta-Glucocerebrosidase Gene Associated with Neurologic Manifestations in Three Sibs. Clin Genet 53:281–285. doi: 10.1034/j.1399-0004.1998.530410.x.
51. Beutler E, Kattamis C, Sipe J, Lipson M (1995) 1342C Mutation in Gaucher's Disease. Lancet 346:1637. doi: 10.1016/S0140-6736(95)92848-0.
52. Balicki D, Beutler E (1995) Gaucher Disease. Medicine (Baltimore) 74:305–323. doi: 10.1097/00005792-199511000-00002
53. Uyama E, Takahashi K, Owada M, et al (1992). Hydrocephalus, corneal opacities, deafness, valvular heart disease, deformed toes and leptomeningeal fibrous thickening in adult siblings: a new syndrome associated with beta-glucocerebrosidase deficiency and a mosaic population of storage cells. Acta Neurol Scand 86:407–420. doi: 10.1111/j.1600-0404.1992.tb05195.x.
54. Hermann G, Wagner LD, Gendal ES, et al (1989). Spinal cord compression in type I Gaucher disease. Radiology 170(1 Pt 1):147–148. doi: 10.1148/radiology.170.1.2744325.
55. D'Amore S, Page K, Donald A, et al; MRC GAUCHERITE Consortium (2021). In-depth phenotyping for clinical stratification of Gaucher disease. Orphanet J Rare Dis 16:431. doi: 10.1186/s13023-021-02093-4.
56. Lopez G, Steward A, Ryan E, et al (2020). Clinical evaluation of sibling pairs with Gaucher disease discordant for parkinsonism. Mov Disord 35:359–365. doi: 10.1002/mds.27973.
57. Steward AM, Wiggs E, Lindstrom T, et al (2019). Variation in cognitive function over time in Gaucher disease type 3. Neurology 93:e2272–83. doi: 10.1212/WNL.0000000000008462.
58. Thaler A, Gurevich T, Bar Shira A, et al (2017). A "dose" effect of mutations in the GBA gene on Parkinson's disease phenotype. Parkinsonism Relat Disord 36:47–51. doi: 10.1016/j.parkreldis.2016.12.019.
59. Devigili G, De Filippo M, Ciana G, et al (2017). Chronic pain in Gaucher disease: skeletal or neuropathic origin? Orphanet J Rare Dis 12:148. doi: 10.1186/s13023-017-0697-1.
60. Alcalay RN, Dinur T, Quinn T, et al (2014). Comparison of Parkinson risk in Ashkenazi Jewish patients with Gaucher disease and GBA heterozygotes. JAMA Neurol 71:752–757. doi: 10.1001/jamaneurol.2014.467.
61. Kuter DJ, Mehta A, Hollak CE, et al (2013). Miglustat therapy in type 1 Gaucher disease: clinical and safety outcomes in a multicenter retrospective cohort study. Blood Cells Mol Dis 51:116–124. doi: 10.1016/j.bcmd.2013.04.001.
62. Kumar KR, Ramirez A, Göbel A, et al (2013). Glucocerebrosidase mutations in a Serbian Parkinson's disease population. Eur J Neurol 20:402–405. doi: 10.1111/ene.12010.
63. Chetrit EB, Alcalay RN, Steiner-Birmanns B, et al (2013). Phenotype in patients with Gaucher disease and Parkinson disease. Blood Cells Mol Dis 50:218–221. doi: 10.1016/j.bcmd.2012.10.009.
64. Stirnemann J, Vigan M, Hamroun D, et al (2012). The French Gaucher's disease registry: clinical characteristics, complications and treatment of 562 patients. Orphanet J Rare Dis 7:77. doi: 10.1186/1750-1172-7-77.
65. Benko W, Ries M, Wiggs EA, et al (2011). The saccadic and neurological deficits in type 3 Gaucher disease. PLoS One 6:e22410. doi: 10.1371/journal.pone.0022410.
66. Rosenbloom B, Balwani M, Bronstein JM, et al (2011). The incidence of Parkinsonism in patients with type 1 Gaucher disease: data from the ICGG Gaucher Registry. Blood Cells Mol Dis 46:95–102. doi: 10.1016/j.bcmd.2010.09.002.
67. Giraldo P, Capablo JL, Alfonso P, et al (2011). Neurological manifestations in patients with Gaucher disease and their relatives, it is just a coincidence? J Inherit Metab Dis 34:781–787. doi: 10.1007/s10545-011-9301-y.
68. Lesage S, Anheim M, Condroyer C, et al (2011). Large-scale screening of the Gaucher's disease-related glucocerebrosidase gene in Europeans with Parkinson's disease. Hum Mol Genet 20:202–210. doi: 10.1093/hmg/ddq454.
69. Biegstraaten M, Mengel E, Maródi L, et al (2010). Peripheral neuropathy in adult type 1 Gaucher disease: a 2-year prospective observational study. Brain 133:2909–2919. doi: 10.1093/brain/awq237.
70. Chérin P, Rose C, de Roux-Serratrice C, et al (2010). The neurological manifestations of Gaucher disease type 1: the French Observatoire on Gaucher disease (FROG). J Inherit Metab Dis 33:331–338. doi: 10.1007/s10545-010-9205-0.
71. Bultron G, Kacena K, Pearson D, et al (2010). The risk of Parkinson's disease in type 1 Gaucher disease. J Inherit Metab Dis 33:167–173. doi: 10.1007/s10545-010-9178-z.
72. Goker-Alpan O, Wiggs EA, Eblan MJ, et al (2008). Cognitive outcome in treated patients with chronic neuronopathic Gaucher disease. J Pediatr 153:89–94. doi: 10.1016/j.jpeds.2007.12.017.
73. Alfonso P, Aznarez S, Giralt M, et al (2007). Spanish Gaucher’s Disease Registry. Mutation analysis and genotype/phenotype relationships of Gaucher disease patients in Spain. J Hum Genet 52:391–396. doi: 10.1007/s10038-007-0113-5.
74. Tylki-Szymañska A, Keddache M, Grabowski GA (2006). Characterization of neuronopathic Gaucher disease among ethnic Poles. Genet Med 8:8–15. doi: 10.1097/01.gim.0000190952.10570.19.
75. Clark LN, Nicolai A, Afridi S, et al (2005). Pilot association study of the beta-glucocerebrosidase N370S allele and Parkinson's disease in subjects of Jewish ethnicity. Mov Disord 20:100–103. doi: 10.1002/mds.20299.
76. Goker-Alpan O, Schiffmann R, LaMarca ME, et al (2004). Parkinsonism among Gaucher disease carriers. J Med Genet 41:937–940. doi: 10.1136/jmg.2004.022772.
77. Mercimek-Mahmutoglu S, Gruber S, Rolfs A, et al (2007). Neurological and brain MRS findings in patients with Gaucher disease type 1. Mol Genet Metab 91:390–395. doi: 10.1016/j.ymgme.2007.03.003.
78. Aharon-Peretz J, Rosenbaum H, Gershoni-Baruch R (2004). Mutations in the glucocerebrosidase gene and Parkinson's disease in Ashkenazi Jews. N Engl J Med 351:1972–1977. doi: 10.1056/NEJMoa033277.
79. Pastores GM, Barnett NL, Bathan P, Kolodny EH (2003). A neurological symptom survey of patients with type I Gaucher disease. J Inherit Metab Dis 26:641–645. doi: 10.1023/A:1025562429898.
80. Tayebi N, Walker J, Stubblefield B, et al (2003). Gaucher disease with parkinsonian manifestations: does glucocerebrosidase deficiency contribute to a vulnerability to parkinsonism? Mol Genet Metab 79:104–109. doi: 10.1016/S1096-7192(03)00047-4.
81. Ida H, Rennert OM, Iwasawa K, et al (1999). Clinical and genetic studies of Japanese homozygotes for the Gaucher disease L444P mutation. Hum Genet 105:120–126. doi: 10.1007/s004390051082.
82. Rice EO, Mifflin TE, Sakallah S, et al (1996). Gaucher disease: studies of phenotype, molecular diagnosis and treatment. Clin Genet 49:111–118. doi: 10.1111/j.1399-0004.1996.tb02728.x.
83. Neudorfer O, Giladi N, Elstein D, et al (1996). Occurrence of Parkinson's syndrome in type 1 Gaucher disease. Q J Med 89:691–694. doi: 10.1093/qjmed/89.9.691.
84. Tylki-Szymańska A, Millat G, dMaire I, Czartoryska B (1996). Types I and III Gaucher disease in Poland: incidence of the most common mutations and phenotypic manifestations. Eur J Hum Genet 4:334–337. doi: 10.1038/sj.ejhg.5200100.
85. Grewal RP, Doppelt SH, Thompson MA, et al (1991). Neurologic complications of nonneuronopathic Gaucher's disease. Arch Neurol 48:1271–1272. doi: 10.1001/archneur.1991.00530240075022.
